# Psychological distress and its potential risk factors among Black and White adult immigrants in the United States – National Health Interview Survey 2005–2018

**DOI:** 10.1016/j.pmedr.2025.103052

**Published:** 2025-04-03

**Authors:** David Adzrago, Maryam Elhabashy, David R. Williams, Faustine Williams

**Affiliations:** a Division of Intramural Research, National Institute on Minority Health and Health Disparities, National Institutes of Health, Bethesda, MD, USA; b Department of Social and Behavioral Sciences, Harvard T. H. Chan School of Public Health, Harvard University, Boston, MA, USA

**Keywords:** Psychological distress, Anxiety, Depression, Mental health, Immigrant health, Health disparities

## Abstract

**Objective:**

Psychological distress is associated with increased likelihood of chronic disease and mortality. Limited research has explored psychological distress among nationally representative minority and immigrant persons, especially Black and White immigrant populations who may be uniquely susceptible to psychological distress, its risk factors, and comorbidities. This current study aimed to estimate the prevalence of moderate to severe (hereafter, moderate-severe) psychological distress and assess its risk factors among Black and White immigrants, respectively.

**Methods:**

Drawing from the 2005–2018 National Health Interview Surveys data, this study analyzed a sample of Black (*n* = 5939) and White (*n* = 40,127) immigrants using weighted Chi-square test and logistic regression analyses. The interaction between race (Black and White immigrants) and each of the predictors was assessed, adjusting for the rest of the predictors.

**Results:**

The prevalence of moderate-severe psychological distress was higher among White immigrants (19.56 %) compared to Black immigrants (17.43 %). Several sociodemographic and behavioral risk factors (i.e., low education, higher BMI, poverty, and smoking) were more strongly associated with elevated distress among Black immigrants. Age, acculturation, and alcohol drinking status significantly moderated the association between race and moderate-severe psychological distress among immigrants.

**Conclusions:**

The findings revealed that psychological distress prevalence and risk factors differ across Black and White immigrant populations, with more pronounced behavioral risks among Black immigrants. More population-specific mental health interventions may help reduce mental health disparities in immigrant communities while conducting longitudinal studies to characterize mental health patterns and changes with their risk factors over time among immigrant populations.

## Highlights

• White immigrants experience more moderate-severe psychological distress than Black immigrants
• Acculturation and alcohol drinking moderate the association between race and distress
• Acculturation is a protective factor for Black immigrants only
• Alcohol drinking is a risk factor for White immigrants only
• Smoking is a risk factor for both races, particularly in Black immigrants

## 1 Introduction

Psychological distress, an array of non-specific symptoms of stress, anxiety, and depression, is a significant public health problem increasing the risk of temporary or permanent health problems encompassing mental health problems (e.g., depression, anxiety) (Brijnath et al., 2019), chronic conditions (e.g., arthritis, lung disease, cardiovascular disease) (McLachlan and Gale, 2018), and mortality (Muhuri, 2014). While a growing body of research investigates differences in psychological distress among Black and White United States (US) adults (Wen et al., 2023; Williams, 2019; Barnes and Bates, 2017; Adzrago et al., 2024), the findings are mixed and require more studies. One systematic review indicates that while some national studies reported higher distress among Black persons compared to White persons, others found lower or no significant differences (Barnes and Bates, 2017). The paradox that Black individuals have worse socioeconomic status but have equal or lower and/or higher distress constitutes a need for data disaggregation and more research.

The Black-White differences in distress may be partly explained by immigration status. However, limited research compares psychological distress among Black and White immigrant adults. The limited existing literature suggests that Black immigrants have a lower prevalence of anxiety and depression than White immigrants (Breslau et al., 2009). Nevertheless, Black and White immigrant adults' varying experiences of acculturation, cultural differences, and racial and/or ethnic discrimination may have unique implications for psychological distress risk factors and prevalence (Alegría et al., 2017; Abboud et al., 2019; Adida and Robinson, 2023). Furthermore, immigrant populations may face additional barriers (e.g., stigma, language proficiency, lack of health insurance) to seeking mental health treatment (Mohammadifirouzeh et al., 2023). According to the minority stress theory, individuals from minority groups—especially those with multiple disadvantaged identities or socioeconomic conditions—often face increased risks of stress and adverse health outcomes (Dohrenwend, 2000; Flentje et al., 2020; Alvidrez et al., 2021). In general, immigrants' mental health tends to decrease with longer length of stay in their destination countries (Ikonte et al., 2020; Lee et al., 2024). This underscores the need to examine mental health disparities among Black and White immigrants, two growing populations in the US (Donnella, 2023; Schneider, 2023). Disaggregating data can make populations more comparable and reduce differences in population characteristics that obscure relationships.

Existing studies have noted sociodemographic and health behavior differences in psychological distress. The risk of experiencing psychological distress is higher among individuals who are younger, females, uninsured, and have lower education and income (Adzrago et al., 2024; Gullett et al., 2022; Association CMH, 2008; Jokela, 2022; Breslau et al., 2021; McGinty et al., 2020). Similarly, divorced/separated/widowed persons, those who are unemployed, obese, and engaging in poor health behaviors such as physical inactivity and substance use (e.g., alcohol, cigarettes) are more likely to experience psychological distress (McLachlan and Gale, 2018; Adzrago et al., 2024; McGinty et al., 2020; Parker et al., 2021; Zvolensky et al., 2018; Ashdown-Franks et al., 2020; Liu and Wang, 2024). The lack of studies investigating psychological distress among Black and White immigrants represents an important research gap. We therefore analyzed national data to (a) estimate the prevalence of psychological distress among Black and White adult immigrants and (b) examine the association of socioeconomic and behavioral risk factors with psychological distress for both Black and White adult immigrants. Exploring the extent to which the correlates of psychological distress among immigrants vary by race can help to better understand the social context of mental health disparities in immigrant communities. This information is essential to inform future research, policy, and treatment initiatives focused on adequately addressing mental health disparities to ensure improved immigrant health in the US.

## 2 Methods

### 2.1 Data sources and sample

Data from the 2005–2018 National Health Interview Survey (NHIS) were analyzed for this study. The NHIS is an annual nationally representative cross-sectional survey among US civilian noninstitutionalized individuals residing within the 50 states and the District of Columbia (Blewett et al., 2023; Keralis et al., 2023; NCHS. National Health Interview Survey, 2018). It assesses mental and physical health conditions, health behaviors (e.g., physical activity, substance use), and sociodemographic information (e.g., age, sex, nativity, race and/or ethnicity) among children aged <17 years and adults aged ≥18 years (Blewett et al., 2023; Keralis et al., 2023; NCHS. National Health Interview Survey, 2018). Stratified, complex clustered sampling techniques are used to select a random sample of dwelling units and participants by partitioning the US into several nested levels of strata and clusters (Keralis et al., 2023; NCHS. National Health Interview Survey, 2018). This current study draws from a pooled sample of 200,693 immigrant adults (i.e., individuals not born in the US) from the 2005–2018 NHIS. Survey data collected beyond 2018 were not included due to the content and structural redesigning of the 2019 NHIS, which included triannual administering of relevant mental health scales as opposed to annual administering. This annual change, among the content and structural redesigning, renders data collected past 2018 incompatible with earlier NHIS data. We further restricted the analysis to immigrant adults (*n* = 55,751) who are Black (*n* = 7182) and White (*n* = 48,569) individuals. Individuals with missing responses for any variables of interest were excluded, and we conducted complete case analyses based on psychological distress among 46,066 immigrant adults who are Black (*n* = 5939) and White (*n* = 40,127) individuals (see Appendix A for detailed information on inclusion and exclusion criteria). NHIS data are publicly available de-identified data and therefore access to the data for our study did not require an Institutional Review Board approval.

### 2.2 Measures

#### 2.2.1 Psychological distress status

The Kessler Psychological Distress Scale (K6) was used to determine psychological distress status among the participants. Participants self-reported to six questions (i.e., K6 scale) regarding how often they felt sad, nervous, restless or fidgety, hopeless, everything was an effort, or worthless in the past 30 days, with responses on a five-point Likert-type rating scale ranging from zero (none of the time) to four (all of the time) (Furukawa et al., 2003). Total scores range from zero to 24, with higher scores indicating higher psychological distress (Furukawa et al., 2003; Forman-Hoffman et al., 2014; Kessler et al., 2003). A total score of five or higher has been used to indicate moderate to severe (henceforth, moderate-severe) psychological distress; otherwise, they indicate no to mild psychological distress (Furukawa et al., 2003; Forman-Hoffman et al., 2014; Kessler et al., 2003).

#### 2.2.2 Predictors of moderate-severe psychological distress

The potential risk factors assessed included a) demographic factors: age (18–25, 26–34, 35–44, 45–54, 55–64, or ≥65), sex (male or female), marital status (divorced, separated, widowed, married/living with a partner, or single/never married); b) socioeconomic factors: employment status (employed or unemployed), level of education completed (less than high school, high school, some college, or ≥ college), poverty status (income below poverty threshold or at/above poverty threshold); c) other sociodemographic factors: region of residence (Northeast, Midwest, South, or West) and acculturation/length of stay in the US (less than 10 years or 10 years or more); and d) health behaviors and related risk factors: health insurance status (insured or uninsured), body mass index (BMI) status (underweight/normal [BMI <25], overweight [BMI 25 to BMI <30], or obese [BMI ≥30]), physical activity status, alcohol drinking status, and cigarette smoking status. The 2018 physical activity guidelines for Americans were used to define physical activity status (HHS, 2023). Alcohol drinking status included non-users/lifetime abstainers (fewer than 12 drinks in their lifetime), former users (at least 12 drinks in any year in their lifetime, but no drinks in the past year), and current users (one to 11 or more drinks in the past year). Smoking status included never users (never smoked 100 cigarettes in their lifetime), former users (ever smoked 100 cigarettes in their lifetime but currently do not smoke cigarettes), and current users (ever smoked 100 cigarettes in their lifetime and currently smoke cigarettes).

### 2.3 Statistical analysis

Sampling weights were used to account for the survey's multistage, complex sampling design and nonresponses to produce accurate and nationally representative population estimates. We first computed unweighted frequencies with their corresponding weighted percentages, using descriptive and bivariate statistics. We used Rao-Scott χ^2^ tests for the bivariate statistics to test differences in the prevalence of psychological distress between groups (i.e., by the predictors). Also, we used three multivariable logistic regression models to examine the association between psychological distress and the predictors; assessing the association among the overall sample of Black and White immigrant adults (Model 1), among Black immigrant adults (Model 2), and among White immigrant adults (Model 3). Furthermore, we assessed the interaction between race (Black and White immigrants) and each of the predictors, adjusting for the rest of the predictors, resulting in 13 models. Odds ratios with 95 % confidence intervals (95 % CIs) were reported as estimates to determine associations computed in the multivariable logistic regression models. Additionally, we examined the interaction effects for the significant interactions by estimating the average predicted probabilities or the overall predictive margins using margins command in Stata and representing the estimates in graphs with margins plots. The level of statistical significance was set as a two-tailed *p* < 0.05. All statistical analyses were conducted using Stata version 18.0.

## 3 Results

### 3.1 Population characteristics

The sociodemographic and behavioral characteristics (except education level) of the participants were similar between the overall immigrant population (both Black and White population), Black immigrants, and White immigrants (Table 1). The majority were 35–44 years old, female, married/living with a partner, employed, had health insurance, and lived in the US for 10 years or more. They mostly lived in the US South, were at or above the poverty threshold, reported being overweight, inactive/insufficiently active, current alcohol drinkers, and never smokers. While the majority of the overall immigrants and White immigrants had similarly less than high school education, the majority of Black immigrants had some college/associate degree or ≥college degree.

**Table 1:** Moderate-severe psychological distress prevalence by sociodemographic and behavioral characteristics of Black and White adult immigrants in the United States (*n* = 46,066), 2005–2018 National Health Interview Survey (NHIS).

	Black and White			Black			White		
	Total Sample	Moderate-Severe Psychological Distress (Yes)		Total Sample	Moderate-Severe Psychological Distress (Yes)		Total Sample	Moderate-severe Psychological Distress (Yes)	
	N (%)	n (%)	*p*-value	N (%)	n (%)	p-value	N (%)	n (%)	p-value
Overall	46,066	8995 (19.28)		5939	1043 (17.43)		40,127	7952 (19.56)	
Age			<0.001			0.65			<0.001
18–25 years old	4181 (9.31)	732 (18.15)		580 (11.19)	111 (19.83)		3601 (9.02)	621 (17.83)	
26–34 years old	9093 (19.65)	1519 (16.68)		1205 (21.07)	213 (16.97)		7888 (19.44)	1306 (16.63)	
35–44 years old	11,108 (22.73)	1991 (17.55)		1421 (23.38)	249 (18.00)		9687 (22.63)	1742 (17.48)	
45–54 years old	8585 (18.27)	1802 (20.11)		1233 (19.98)	211 (16.69)		7352 (18.01)	1591 (20.69)	
55–64 years old	5888 (13.23)	1358 (22.27)		754 (12.42)	129 (16.23)		5134 (13.35)	1229 (23.13)	
≥65 years old	7211 (16.81)	1593 (22.03)		746 (11.97)	130 (17.36)		6465 (17.55)	1463 (22.52)	
Sex			<0.001			<0.001			<0.001
Female	24,984 (52.14)	5715 (22.68)		3204 (51.76)	640 (20.65)		21,780 (52.20)	5075 (22.98)	
Male	21,082 (47.86)	3280 (15.58)		2735 (48.24)	403 (13.97)		18,347 (47.80)	2877 (15.83)	
Acculturation			<0.001			0.82			<0.001
Less than 10 years	9430 (20.44)	1581 (16.79)		1469 (25.71)	261 (17.21)		7961 (19.64)	1320 (16.71)	
10 years or more	36,636 (79.56)	7414 (19.92)		4470 (74.29)	782 (17.51)		32,166 (80.36)	6632 (20.26)	
Marital status			<0.001			<0.001			<0.001
Divorced	5080 (11.31)	1284 (25.39)		825 (14.16)	176 (21.49)		4255 (10.88)	1108 (26.16)	
Separated	2576 (4.94)	733 (27.06)		428 (6.90)	101 (20.81)		2148 (4.64)	632 (28.47)	
Widowed	2970 (6.79)	784 (26.04)		290 (4.35)	64 (23.36)		2680 (7.16)	720 (26.29)	
Married/living with partner	26,760 (57.77)	4389 (16.14)		2768 (47.10)	374 (13.13)		23,992 (59.38)	4015 (16.51)	
Single/Never married	8680 (19.19)	1805 (20.73)		1628 (27.49)	328 (20.92)		7052 (17.93)	1477 (20.68)	
Region of residence			<0.001			0.24			<0.001
Northeast	9372 (22.25)	1986 (20.16)		2139 (36.09)	390 (18.41)		7233 (20.15)	1596 (20.64)	
North Central/Midwest	4873 (11.87)	993 (20.35)		665 (11.65)	123 (17.20)		4208 (11.90)	870 (20.82)	
South	16,680 (36.35)	3024 (17.60)		2426 (41.65)	398 (16.05)		14,254 (35.55)	2626 (17.87)	
West	15,141 (29.53)	2992 (20.26)		709 (10.61)	132 (19.78)		14,432 (32.40)	2860 (20.29)	
Employment status			<0.001			<0.001			<0.001
Employed	28,877 (63.24)	4453 (15.41)		4107 (70.06)	619 (15.00)		24,770 (62.20)	3834 (15.48)	
Not employed	17,189 (36.76)	4542 (25.94)		1832 (29.94)	424 (23.13)		15,357 (37.80)	4118 (26.27)	
Health insurance status			0.19			0.07			0.03
Insured	30,922 (71.18)	6149 (19.46)		4551 (77.96)	780 (16.85)		26,371 (70.15)	5369 (19.90)	
Uninsured	15,144 (28.82)	2846 (18.84)		1388 (22.04)	263 (19.49)		13,756 (29.85)	2583 (18.77)	
Education			<0.001			<0.001			<0.001
Less than high school	16,827 (30.84)	3734 (21.98)		1128 (16.63)	251 (21.33)		15,699 (33.00)	3483 (22.03)	
High school graduate	10,356 (22.20)	1964 (19.20)		1441 (23.65)	278 (19.18)		8915 (21.98)	1686 (19.21)	
Some college/Associate degree	9368 (21.67)	1833 (19.80)		1748 (29.97)	311 (18.66)		7620 (20.42)	1522 (20.06)	
≥College degree	9515 (25.29)	1464 (15.62)		1622 (29.75)	203 (12.62)		7893 (24.60)	1261 (16.17)	
Poverty status			<0.001			<0.001			<0.001
Below poverty threshold	11,905 (22.81)	3230 (27.31)		1412 (22.56)	356 (25.34)		10,493 (22.85)	2874 (27.60)	
At or above poverty threshold	34,161 (77.19)	5765 (16.91)		4527 (77.44)	687 (15.13)		29,634 (77.15)	5078 (17.18)	
Body mass index (BMI)			<0.001			<0.001			<0.001
Underweight/Normal (BMI <25)	15,654 (35.23)	2799 (17.66)		2052 (34.75)	330 (16.01)		13,602 (35.30)	2469 (17.91)	
Overweight (BMI ≥25 & BMI <30)	18,506 (39.95)	3316 (17.78)		2401 (40.57)	380 (15.18)		16,105 (39.85)	2936 (18.18)	
Obese (BMI ≥30)	11,906 (24.83)	2880 (23.99)		1486 (24.68)	333 (23.12)		10,420 (24.85)	2547 (24.12)	
Physical activity			0.49			0.81			0.42
Inactive/Insufficient	45,327 (98.19)	8853 (19.30)		5830 (97.93)	1022 (17.41)		39,497 (98.23)	7831 (19.59)	
Physically active	739 (1.81)	142 (18.14)		109 (2.07)	21 (18.35)		630 (1.77)	121 (18.10)	
Alcohol drinking status			<0.001			0.005			<0.001
Never	15,358 (31.00)	2965 (19.18)		2426 (39.79)	427 (18.16)		12,932 (29.67)	2538 (19.39)	
Former	5848 (12.05)	1462 (25.25)		635 (10.33)	132 (21.78)		5213 (12.31)	1330 (25.69)	
Current	24,860 (56.95)	4568 (18.07)		2878 (49.88)	484 (15.95)		21,982 (58.02)	4084 (18.35)	
Smoking status			<0.001			<0.001			<0.001
Never	33,206 (70.34)	5913 (17.49)		4855 (82.19)	793 (16.25)		28,351 (68.54)	5120 (17.72)	
Former	7584 (17.87)	1648 (21.38)		592 (9.70)	117 (19.99)		6992 (19.11)	1531 (21.49)	
Current	5276 (11.79)	1434 (26.78)		492 (8.11)	133 (26.38)		4784 (12.35)	1301 (26.82)	

Notes: Frequencies are unweighted while percentages are weighted using the 2005-2018 NHISs sampling weights. We used Rao-Scott χ^2^ tests to determine the statistically significant estimates. The difference in the prevalence of moderate-severe psychological distress between Black and White immigrants was statistically significant (*p* = 0.003). We used the Kessler Psychological Distress Scale (K6) to measure psychological distress, with total self-reported K6 scores of ≥5 out of 24 indicating moderate-severe psychological distress.

### 3.2 Prevalence of moderate-severe psychological distress

Overall, immigrants (19.28 %) and White immigrants (19.56 %) similarly reported higher prevalence of moderate-severe psychological distress compared to Black immigrants (17.43 %) (Table 1). There were similarities and differences in the prevalence between the overall immigrants, Black immigrants, and White immigrants. Among the overall immigrants, Black immigrants, and White immigrants, individuals who were female, unemployed, had less than high school education, lived below the income poverty threshold, obese, former alcohol drinkers, and current smokers had elevated prevalence of moderate-severe psychological distress. All these differences in the prevalence within the groups were statistically significant. Among only the overall and White immigrants, those who were aged 55–64, lived in the US for at least 10 years, and resided in the Midwest reported higher prevalence. Having health insurance was associated with the prevalence of distress among only White immigrants. Physical activity was not associated with the prevalence across the overall immigrants, Black immigrants, and White immigrants.

### 3.3 Factors associated with psychological distress

Age, sex, acculturation, marital status, employment status, education, income poverty status, BMI, alcohol drinking status, and smoking status were significantly associated with moderate-severe psychological distress, while race, region of residence, health insurance status, and physical activity were not significantly associated with moderate-severe psychological distress (Table 2: Model 1). Compared to those aged 18–25, higher odds of moderate-severe psychological distress were observed for individuals aged 45–54 (OR = 1.22; 95 % CI = 1.06, 1.41) and 55–64 (OR = 1.89; 95 % CI = 1.03, 1.37), while lower odds were observed for those aged ≥65 (OR = 0.86; 95 % CI = 0.74, 0.99). Females had higher odds (OR = 1.51; 95 % CI = 1.41, 1.61) compared to males. Those who lived in the US less than 10 years had lower odds (OR = 0.91; 95 % CI = 0.84, 0.99) relative to those present for 10 years or more. Compared to those single/never married, higher odds were found among those who were separated (OR = 1.14; 95 % CI = 1.02, 1.29), while lower odds were observed for married/living with a partner individuals (OR = 0.71; 95 % CI = 0.65, 0.77). Unemployment (vs. being employed), higher education (vs. less than high school), overweight/obese (vs. underweight/normal weight), former/current alcohol drinking (vs. never used), and former/current smoking (vs. never smoked) were all significantly associated with higher odds of moderate-severe psychological distress.

**Table 2:** Multivariable logistic regression analysis of moderate-severe psychological distress and its risk factors in Black and White adult immigrants in the United States, 2005–2018 National Health Interview Survey (NHIS).

	Model 1: Black and White	Model 2: Black	Model 3: White
	OR (95 % CI)	OR (95 % CI)	OR (95 % CI)
Race			
Black	0.94 (0.85, 1.03)	**–**	**–**
White	1.00	**–**	**–**
Age			
18–25 years old	1.00	1.00	1.00
26–34 years old	1.04 (0.91, 1.20)	1.03 (0.74, 1.42)	1.05 (0.90, 1.21)
35–44 years old	1.09 (0.95, 1.24)	1.07 (0.78, 1.47)	1.09 (0.94, 1.25)
45–54 years old	1.22 (1.06, 1.41)	0.96 (0.67, 1.37)	1.27 (1.09, 1.47)
55–64 years old	1.89 (1.03, 1.37)	0.79 (0.56, 1.12)	1.25 (1.07, 1.46)
≥65 years old	0.86 (0.74, 0.99)	0.63 (0.43, 0.94)	0.89 (0.75, 1.04)
Sex			
Female	1.51 (1.41, 1.61)	1.47 (1.22, 1.78)	1.51 (1.40, 1.62)
Male	1.00	1.00	1.00
Acculturation			
Less than 10 years	0.91* (0.84, 0.99)	0.98 (0.76, 1.19)	0.89** (0.82, 0.97)
10 years or more	1.00	1.00	1.00
Marital status			
Divorced	1.11 (0.99, 1.24)	1.06 (0.82, 1.37)	1.13 (1.01, 1.28)
Separated	1.14 (1.02, 1.29)	0.91 (0.68, 1.22)	1.21 (1.06, 1.38)
Widowed	0.99 (0.87, 1.13)	1.05 (0.71, 1.55)	0.99 (0.87, 1.14)
Married/living with partner	0.71 (0.65, 0.77)	0.63 (0.52, 0.77)	0.72 (0.66, 0.79)
Single/Never married	1.00	1.00	1.00
Region of residence			
Northeast	1.00	1.00	1.00
North Central/Midwest	1.10 (0.98, 1.24)	0.99 (0.74, 1.33)	1.11 (0.98, 1.25)
South	0.92 (0.84, 1.00)	0.95 (0.76, 1.18)	0.91 (0.83, 0.99)
West	1.07 (0.98, 1.17)	1.18 (0.85, 1.64)	1.06 (0.96, 1.16)
Employment status			
Employed	1.00	1.00	1.00
Not employed	1.72 (1.61, 1.84)	1.50 (1.24, 1.82)	1.75 (1.63, 1.88)
Health insurance status			
Insured	1.04 (0.97, 1.12)	0.95 (0.76, 1.19)	1.05 (0.97, 1.13)
Uninsured	1.00	1.00	1.00
Education			
Less than high school	1.16 (1.06, 1.27)	1.28 (0.97, 1.68)	1.15 (1.05, 1.26)
High school graduate	1.09 (1.00, 1.20)	1.35 (1.06, 1.71)	1.06 (0.96, 1.17)
Some college/Associate degree	1.12 (1.02, 1.24)	1.29 (1.02, 1.63)	1.09 (0.98, 1.21)
≥College degree	1.00	1.00	1.00
Poverty status			
Below poverty threshold	1.00	1.00	1.00
At or above poverty threshold	0.67 (0.63, 0.72)	0.72 (0.60, 0.88)	0.66 (0.62, 0.71)
Body mass index (BMI)			
Underweight/Normal (BMI <25)	1.00	1.00	1.00
Overweight (BMI ≥25 & BMI <30)	1.08 (1.01, 1.16)	1.08 (0.89, 1.31)	1.08 (1.01, 1.17)
Obese (BMI ≥30)	1.40 (1.29, 1.51)	1.58 (1.29, 1.94)	1.38 (1.27, 1.51)
Physical activity			
Inactive/Insufficient	1.00	1.00	1.00
Physically active	0.96 (0.77, 1.19)	1.15 (0.68, 1.93)	0.93 (0.73, 1.18)
Alcohol drinking status			
Never	1.00	1.00	1.00
Former	1.38 (1.25, 1.52)	1.14 (0.87, 1.48)	1.43 (1.29, 1.58)
Current	1.19 (1.04, 1.20)	0.88 (0.73, 1.07)	1.17 (1.08, 1.26)
Smoking status			
Never	1.00	1.00	1.00
Former	1.35 (1.24, 1.46)	1.56 (1.19, 2.06)	1.32 (1.21, 1.43)
Current	1.83 (1.68, 1.99)	1.98 (1.51, 2.59)	1.81 (1.65, 1.98)

Notes: Wald's tests were used to determine statistically significant results. The Kessler Psychological Distress Scale (K6) was used to measure psychological distress, with total self-reported K6 scores of ≥5 out of 24 indicating moderate-severe psychological distress.

When stratified by race (Table 2), the odds of moderate-severe psychological distress also varied within Black and White immigrants. For instance, among Black immigrants (Model 2), those aged ≥65 years had lower odds compared to those aged 18–25 years; among White immigrants (Model 3), those aged 45–54 or 55–64 years had higher odds compared to those aged 18–25 years. The odds were lower among White immigrants who lived in the US for less than 10 years (vs. 10 years or more) or resided in the South (vs. Northeast), with no such significant difference among Black immigrants. Higher odds were observed for White divorced or separated immigrants (vs. single/never married), with no such significant pattern among Black immigrants. In contrast with White immigrants with less than high school education, Black immigrants with high school or some college/associate degree had higher odds of moderate-severe psychological distress compared to those with ≥college degree.

### 3.4 Interaction between race and each risk factors among black and white immigrants

**Fig. 1:**
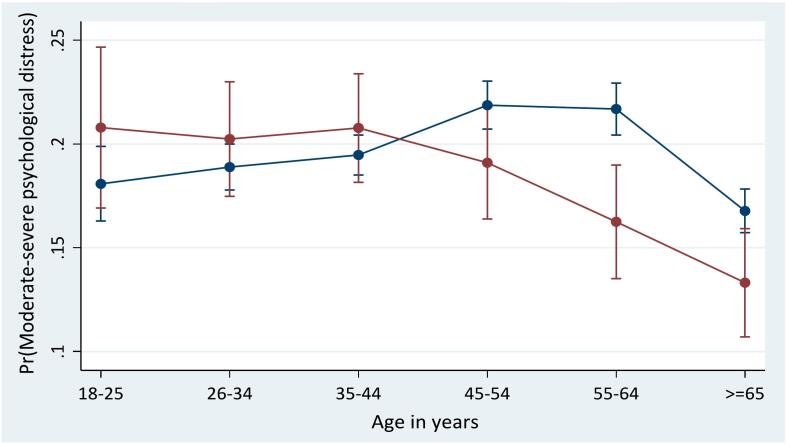

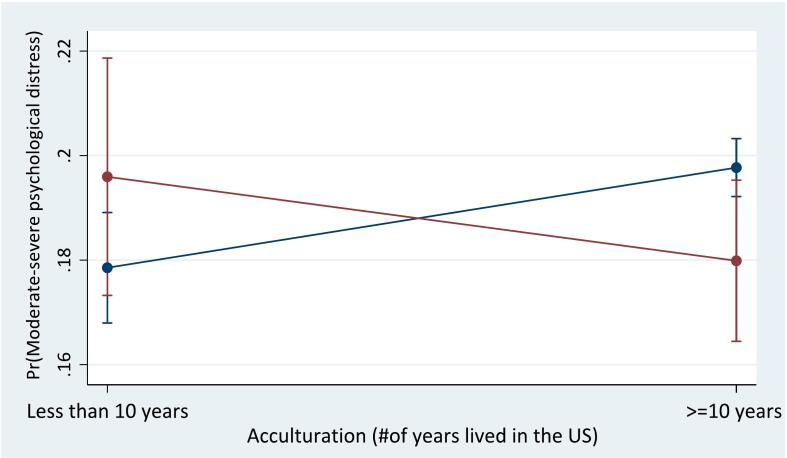

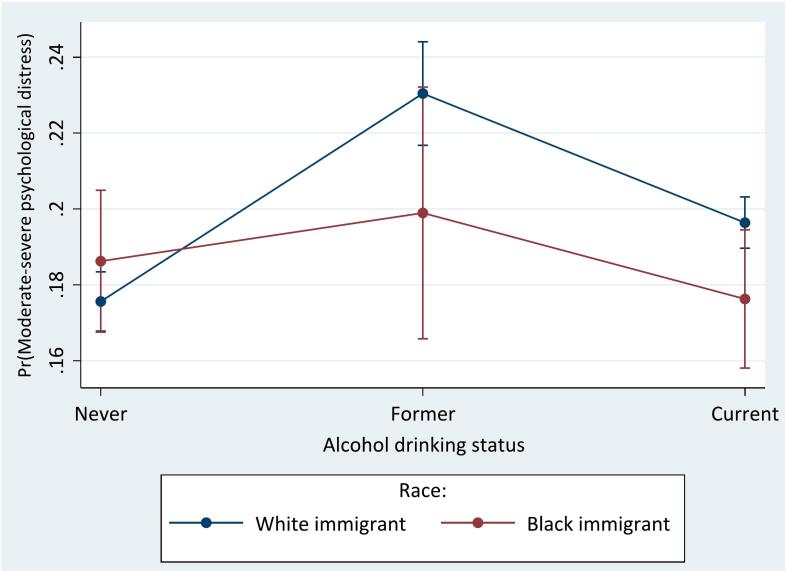
a–c. Predictive margins or average predicted probability (with 95 % CIs) of moderate-severe psychological distress for each level of interaction of risk factors and race among Black and White adult immigrants in the United States. Note: The Kessler Psychological Distress Scale (K6) was used to measure psychological distress, with total self-reported K6 scores of ≥5 out of 24 indicating moderate-severe psychological distress.

The interaction between race and each predictor is presented in Appendix B. There were significant interactions between race and age (*p* = 0.002), acculturation (*p* = 0.02), and alcohol drinking status (*p* = 0.03). Compared to White immigrants aged 18–25, Black immigrants aged 45–54 (OR = 0.69; 95 % CI = 0.50, 0.97), 55–64 (OR = 0.57; 95 % CI = 0.41, 0.80), and ≥65 years (OR = 0.63; 95 % CI = 0.44, 0.89) had lower odds of moderate-severe psychological distress. As displayed in Fig. 1a, White immigrants aged 45–54 years (21.9 %, *p* < 0.001) or 55–64 years (21.7 %, p < 0.001) had the highest probability of moderate-severe psychological distress, while Black immigrants aged ≥65 years had the lowest probability (13.3 %, *p* < 0.001). See Fig. 1a-c for details.

For the interaction between race and acculturation, the odds were higher for Black immigrants who lived in the US less than 10 years (OR = 1.28; 95 % CI = 1.05, 1.55) compared to White immigrants who lived in the US 10 years or more. Fig. 1b shows that White immigrants who lived in the US 10 years or more (19.8 %, *p* < 0.001) and Black immigrants who lived in the US less than 10 years (19.6 %, p < 0.001) similarly had the highest probabilities, while White immigrants who lived in the US less than 10 years (17.9 %, *p* < 0.001) had the lowest probability, followed by Black immigrants who lived in the US 10 years or more (18 %, *p* < 0.001).

Black immigrants who were former (OR = 0.76; 95 % CI = 0.58, 0.99) or current (OR = 0.81; 95 % CI = 0.67, 0.96) alcohol drinkers had lower odds of moderate-severe psychological distress compared to White immigrants who had never used alcohol. As shown in Fig. 1c, in general, White immigrants who were former alcohol drinkers had the highest probability of moderate-severe psychological distress (23 %, *p* < 0.001), while Black immigrants who were current drinkers (17.6 %, p < 0.001) and White immigrants who had never used alcohol (17.6 %, p < 0.001) had the lowest probabilities.

## 4 Discussion

Psychological distress can contribute to or exacerbate various physical and mental health problems (Brijnath et al., 2019; McLachlan and Gale, 2018), which may explain its significant associations with chronic disease and mortality (McLachlan and Gale, 2018; Muhuri, 2014). However, limited research investigates the risk factors or prevalence of psychological distress in various minority communities, especially among Black and White immigrants (Donnella, 2023; Schneider, 2023). Using data from a national survey, we estimated the prevalence of moderate-severe psychological distress and analyzed the relationships between various sociodemographic and behavioral risk factors for moderate-severe psychological distress among Black and White immigrants.

Our study provides evidence of varied patterns of psychological distress among Black and White immigrant populations. We found a higher prevalence and odds (though not statistically significant) of psychological distress among White immigrants compared to Black immigrants. This finding suggests that Black immigrants may have better mental health outcomes than their White immigrant peers (Breslau et al., 2009; Read and Emerson, 2005). For instance, previous research has shown that Black immigrants, especially those from African and Caribbean regions, have lower prevalence of anxiety and mood disorders than White immigrants, particularly those from Western Europe (Breslau et al., 2009). Nonetheless, all Black immigrants may not have better mental health outcomes, including psychological distress, than White immigrants. For example, we identified several characteristics, including obesity, smoking, lower educational attainment, and living below the poverty threshold, that were associated with more elevated risks of experiencing psychological distress among Black immigrants than among White immigrants. Additionally, we observed significant interactions between race and three risk factors: age, acculturation, and alcohol drinking status. These findings emphasize the diversity within the immigrant groups and underscore the importance of considering race as a significant determinant of disparities in psychological distress (Wen et al., 2023; Williams, 2019), as immigrants are not monolithic groups. Understanding how these complex, multifaceted risk factors relate to each other and combine to affect mental health risk is crucial for developing comprehensive and tailored mental health interventions for immigrant populations.

Similar to previous studies, our results showed that regardless of race, the historically disadvantaged socio-demographic and -economic characteristics of being female, unemployed, and living below the poverty threshold put individuals at higher risk of experiencing psychological distress (Stafford et al., 2010; Kirmayer et al., 2011). Within the overall sample, there was also a higher prevalence of moderate-severe psychological distress among individuals who had less than a high school education, were obese, former drinkers, or current smokers. It is possible that behavioral health problems and lower socioeconomic conditions negatively interfere with daily life and cognitive control or neurotransmitters (i.e., dopamine, serotonin, norepinephrine, glutamate) resulting in increased risks of psychological distress among immigrants (Richter and Dixon, 2023; Alegría et al., 2018). Thus, disadvantaged populations likely have greater risks of daily, cumulative, or chronic stress with increased risk of mental health outcomes due to persistent exposure to poor quality conditions (e.g., poor housing, overcrowding, food insecurity, lack of health resources and transportation, discrimination) (Alegría et al., 2018).

While our findings aligned with past studies which observed sex, unemployment, poverty, and obesity as risk factors for psychological distress regardless of race (McLachlan and Gale, 2018; Adzrago et al., 2024; Gullett et al., 2022; Association CMH, 2008; Jokela, 2022; Breslau et al., 2021; McGinty et al., 2020; Parker et al., 2021; Zvolensky et al., 2018), our results further showed that among White immigrants specifically, being overweight was positively associated with experiencing psychological distress. This association may be attributed to perceived weight and beauty standards and the tendency for White individuals, especially females, to experience body dissatisfaction (Ciciurkaite and Perry, 2018). It should be noted that we did not find significant interactions between these factors (sex, employment, income poverty, and BMI) and race but found within-racial group differences. Thus, data disaggregation is necessary in identifying and delineating underlying patterns in mental health outcomes within population subgroups.

Educational attainment did not moderate the association between race and psychological distress in this study. However, we found that educational attainment was significantly associated with psychological distress within Black and White immigrants, respectively: lower levels of educational attainment posed greater risk within Black immigrants than within White immigrants. These findings add to an increasingly nuanced body of research which has generally posited that lower levels of socioeconomic status, such as educational attainment, are associated with higher risks of mental health problems, including psychological distress (Adzrago et al., 2024). One US study found that among Black and White individuals, higher educational attainment was a protective factor against suicide attempts or deaths for White individuals alone (Assari et al., 2019). These findings highlight the importance of data disaggregation and considering unique characteristics of respective populations that may impact immigrant mental health over time.

Further, our study found racial differences in psychological distress to be significantly moderated by age and acculturation. Past research has posited that Black young adults are more likely to experience psychological distress compared to their White counterparts (Tran et al., 2015), which is consistent with our findings. However, our findings do not align with past research indicating that US Black adults experience more psychological distress than White adults (Wen et al., 2023). This inconsistency within Black and White immigrant populations may be related to differences in the acculturation process. For instance, we found that among White immigrants, as acculturation increased, so did the risk of experiencing psychological distress. Conversely, as Black immigrants' acculturation increased, the risk of experiencing psychological distress decreased. These findings regarding age and acculturation may reflect what has previously been described as the ethno-racial context within which immigrants integrate into American society (Hamilton and Hagos, 2021), suggesting that immigrants of particular racial and/or ethnic groups are more likely to experience health outcomes similar to those of their native-born counterparts over time. Since some studies among native-born US adults have shown that White adults are more likely to meet criteria for depression or anxiety symptoms compared to Black adults (Erving et al., 2019), one explanation for our findings could be that as White immigrants age and acculturate into US culture, their experiences of mental health symptoms and coping strategies may begin to mimic those of their native-born counterparts. However, given the inconclusive nature of findings related to psychological distress by race, this relationship warrants more research.

Alcohol drinking status also significantly modulated the racial disparities in psychological distress. In general, White immigrant former or current alcohol drinkers, especially former drinkers, were more likely to experience moderate-severe psychological distress than their Black counterparts. However, Black immigrant current drinkers and White immigrant non-drinkers had similarly decreased odds of experiencing moderate-severe psychological distress. Stratified by race, the results revealed that alcohol drinking behaviors were a significant risk factor among White immigrants, with no such association observed among Black immigrants. Existing research largely examines unidirectional relationships between alcohol intake and psychological distress, with evidence suggesting that psychological distress correlates with increased alcohol intake (Alpers et al., 2023; Halladay et al., 2023). Our findings, however, suggest a potential bidirectional relationship, where alcohol intake may also pose a significant risk for psychological distress (Viertiö et al., 2021). This aligns with past research indicating that while first-generation White (European) immigrants have the highest prevalence of alcohol use disorders, Black immigrants have the lowest rate (Salas-Wright et al., 2015).

Smoking behavior was not a moderator between race and psychological distress in this study. However, we observed that smoking behavior was a significant risk factor for psychological distress within both Black and White immigrants, with more pronounced effects among Black immigrants. While prior research shows that Black individuals in general are less likely to use alcohol and tobacco or nicotine products compared to White individuals (SAMHSA Administion, 2022), there is limited research examining these differences in Black and White immigrants. Besides, immigrants or Black individuals are often studied as one large group, which can obscure within-group disparities or associations. Our findings can support public health research and intervention efforts aimed at addressing mental health needs of racial and/or ethnic minority populations such as Black and White immigrants based on their sociodemographic and behavioral characteristics.

This study has the following limitations. First, because NHIS data is cross-sectional, causal inferences cannot be made. Second, because survey responses were self-reported, results may have been impacted by respondents' social desirability and recall biases. Third, individuals' length of years in the US can be confounded by their age at migration, which can influence their mental health status. For example, immigrants with the same length of stay in the US might have different ages at arrival with varying socioeconomic and mental health experiences. Future research should assess how age at migration and length of stay in the US can independently and jointly influence mental health within various immigrant populations. The incompatibility of NHIS data collected before and from 2019 may also limit the generalizability of this study's findings to the current Black and White immigrant population. Finally, while we considered many factors in the analyses, residual confounding factors (e.g., English proficiency, country of origin) might have further influenced the observed associations.

## 5 Conclusions

Overall, this study found that while moderate-severe psychological distress is evident in both Black and White immigrant populations, White immigrants had higher prevalence than Black immigrants. Moreover, evidence in this study indicates that while there are some shared characteristics that may increase the prevalence and risks of psychological distress among immigrants, there are several characteristics which are more strongly associated with being a Black immigrant (i.e., education, poverty, BMI, and smoking status) or White immigrant (i.e., marital status, employment status, sex, age, and alcohol use). Notably, interaction tests revealed significant racial differences in psychological distress by age, acculturation, and alcohol drinking. Since psychological distress can increase susceptibility to multiple chronic physical and mental health conditions (Brijnath et al., 2019; McLachlan and Gale, 2018), it is imperative that more research explores psychological distress as both a risk factor and health outcome among various immigrant populations. Health education, communication, and mental health resources can be better tailored to the needs of these specific communities, increasing the opportunity to better understand and protect immigrants' health.

## CRediT authorship contribution statement

**David Adzrago:** Writing – review & editing, Writing – original draft, Visualization, Validation, Methodology, Formal analysis, Data curation, Conceptualization. **Maryam Elhabashy:** Writing – review & editing, Writing – original draft, Visualization. **David R. Williams:** Writing – review & editing, Visualization, Methodology. **Faustine Williams:** Writing – review & editing, Visualization, Supervision, Software, Resources, Project administration, Methodology, Conceptualization.

## Ethics approval and consent to participate

Deidentified public-use secondary data were analyzed and therefore no protocol approval was required.

## Funding

This work is supported by the Division of Intramural Research, National Institute on Minority Health and Health Disparities (ZIA MD000015). Opinions and comments expressed in this article belong to the authors and do not necessarily reflect those of the US Government, Department of Health and Human Services, National Institutes of Health, and National Institute on Minority Health and Health Disparities.

## Declaration of competing interest

The authors declare that they have no known competing financial interests or personal relationships that could have appeared to influence the work reported in this paper.

## Data Availability

The datasets generated for the study are publicly available in the CDC database repository, https://www.cdc.gov/nchs/nhis/data-questionnaires-documentation.htm.

## Appendix A Supplementary data

Supplementary material 1 Image 1

Supplementary material 2 Image 2
