# Supplementary material for: Psychological distress and its potential risk factors among Black and White adult immigrants in the United States – National Health Interview Survey 2005–2018
Source: Prev Med Rep. 2025 Apr 3;54:103052. doi: 10.1016/j.pmedr.2025.103052 (PMC12182260; doi:10.1016/j.pmedr.2025.103052)
Supplement: Supplementary file 2 — Supplementary material 2 [file mmc2.docx]

**Appendix A:** Flowchart illustrating criteria for psychological distress among Black and White adult immigrants’ inclusion and exclusion, National Health Interview Survey (NHIS) 2005-2018.

Total United States Adult Population

N= 925,045

Starting Total Immigrant Population

N= 200,693

Excluded (724,352)

- Non-Immigrant

**Total Complete Case Analysis**

**n= 46,066**

**Black**

**n= 5,939**

**White**

**n= 40,127**

Remaining Total Included in Analysis

n= 55,751

Black [n= 7,182]; White [n= 48,569]

Excluded (144,942)

- Non-Black Immigrant
- Non-White Immigrant

Excluded (9,685)

- Missingness
  - Kessler 6 Scale (1,280)
  - Acculturation (810)
  - Health insurance status (185)
  - Marital status (159)
  - Employment status (38)
  - Education (682)
  - Poverty status (5,964)
  - Body mass index (2,434)
  - Alcohol drinking status (1,012)

**Appendix B.** Interaction effects of race and other predictors on moderate-severe psychological distress among Black and White adult immigrants in the United States

| Model A: Race X age | | Model B: Race X sex | | Model C: Race X acculturation | | Model D: Race X marital status | |
| --- | --- | --- | --- | --- | --- | --- | --- |
|  | OR (95% CI) |  | OR (95% CI) |  | OR (95% CI) |  | OR (95% CI) |
| *Main effects*: |  | *Main effects*: |  | *Main effects*: |  | *Main effects*: |  |
| Race |  | Race |  | Race |  | Race |  |
| Black | 1.20 (0.92, 1.56) | Black | 0.94 (0.82, 1.09) | Black | 0.88 (0.79, 0.99) | Black | 1.10 (0.93, 1.29) |
| White | 1.00 | White | 1.00 | White | 1.00 | White | 1.00 |
|  |  |  |  |  |  |  |  |
| Age |  | Sex |  | Acculturation |  | ­ Marital status |  |
| 18-25 years old | 1.00 | Female | 1.51 (1.40, 1.62) | Less than 10 years | 0.88 (0.80, 0.96) | Divorced | 1.16 (1.03, 1.31) |
| 26-34 years old | 1.06 (0.91, 1.23) | Male | 1.00 | 10 years or more | 1.00 | Separated | 1.22 (1.07, 1.39) |
| 35-44 years old | 1.10 (0.96, 1.27) |  |  |  |  | Widowed | 1.03 (0.90, 1.17) |
| 45-54 years old | 1.29 (1.11, 1.49) |  |  |  |  | Married/living with partner | 0.73 (0.67, 0.80) |
| 55-64 years old | 1.27 (1.09, 1.49) |  |  |  |  | Single/Never married | 1.00 |
| ≥65 years old | 0.91 (0.78, 1.06) |  |  |  |  |  |  |
| *Interaction effects*: | F(5, 1114)= 3.90, p= 0.002 | *Interaction effects*: | F(1, 1118)= 0.02, p= 0.90 | *Interaction effects*: | F(1, 1118)= 5.99, p= 0.02 | *Interaction effects*: | F(4, 1115)= 1.84, p= 0.12 |
| Black X 26-34 years old | 0.91 (0.65, 1.28) | Black X female | 0.99 (0.83, 1.17) | Black X less than 10 years | 1.28 (1.05, 1.55) | Black X divorced | 0.80 (0.61, 1.05) |
| Black X 35-44 years old | 0.91 (0.66, 1.24) | White X male | 1.00 | White X 10 years or more | 1.00 | Black X separated | 0.68 (0.49, 0.94) |
| Black X 45-54 years old | 0.69 (0.50, 0.97) |  |  |  |  | Black X widowed | 0.80 (0.55, 1.16) |
| Black X 55-64 years old | 0.57 (0.41, 0.80) |  |  |  |  | Black X married/living with partner | 0.83 (0.68, 1.02) |
| Black X ≥65 years old | 0.63 (0.44, 0.89) |  |  |  |  | White X single/never married | 1.00 |
| White X 18-25 years old | 1.00 |  |  |  |  |  |  |
|  |  |  |  |  |  |  |  |
|  |  |  |  |  |  |  |  |
| Model E: Race X region of residence |  | Model F: Race X employment status |  | Model G: Race X health insurance status |  | Model H: Race X education |  |
| *Main effects:* |  | *Main effects:* |  | *Main effects:* |  | *Main effects:* |  |
| Race |  | Race |  | Race |  | Race |  |
| Black | 0.89 (0.74, 1.07) | Black | 1.00 (0.90, 1.12) | Black | 1.09 (0.90, 1.32) | Black | 0.81 (0.67, 0.99) |
| White | 1.00 | White | 1.00 | White | 1.00 | White | 1.00 |
|  |  |  |  |  |  |  |  |
| Region of residence |  | Employment status |  | Health insurance status |  | Education |  |
| North Central/Midwest | 1.10 (0.97, 1.24) | Not employed | 1.76 (1.64, 1.88) | Insured | 1.06 (0.99, 1.15) | Less than high school | 1.15 (1.04, 1.26) |
| South | 0.91 (0.83, 0.99) | Employed | 1.00 | Uninsured | 1.00 | High school graduate | 1.06 (0.96, 1.17) |
| West | 1.05 (0.96, 1.16) |  |  |  |  | Some college/Associate degree | 1.09 (0.98, 1.21) |
| Northeast | 1.00 |  |  |  |  | >College degree | 1.00 |
|  |  |  |  |  |  |  |  |
| *Interaction effects*: | F(3, 1116)= 0.40, p= 0.75 | *Interaction effects*: | F(1, 1118)= 3.75, p= 0.05 | *Interaction effects*: | F(1, 1118)= 3.30, p= 0.07 | *Interaction effects*: | F(3, 1116)= 2.06, p= 0.10 |
| Black X North Central/Midwest | 0.99 (0.73, 1.36) | Black X not employed | 0.84 (0.70, 1.01) | Black X insured | 0.82 (0.66, 1.02) | Black X less than high school | 1.06 (0.81, 1.39) |
| Black X South | 1.09 (0.87, 1.36) | White X employed | 1.00 | White X uninsured | 1.00 | Black X high school graduate | 1.30 (1.01, 1.67) |
| Black X West | 1.17 (0.83, 1.65) |  |  |  |  | Black X some college/Associate degree | 1.24 (0.96, 1.59) |
| White X Northeast | 1.00 |  |  |  |  | White X >college degree | 1.00 |
|  |  |  |  |  |  |  |  |
|  |  |  |  |  |  |  |  |
| Model I: Race X poverty status |  | Model J: Race X Body mass index |  | Model K: Race X physical activity |  | Model L: Race X alcohol drinking status |  |
| *Main effects*: |  | *Main effects*: |  | *Main effects*: |  | *Main effects*: |  |
| Race |  | Race |  | Race |  | Race |  |
| Black | 0.91 (0.77, 1.08) | Black | 0.96 (0.82, 1.12) | Black | 0.93 (0.85, 1.03) | Black | 1.08 (0.94, 1.24) |
| White | 1.00 | White | 1.00 | White | 1.00 | White | 1.00 |
|  |  |  |  |  |  |  |  |
| Poverty status |  | Body mass index |  | Physical activity |  | Alcohol drinking status |  |
| At or above poverty threshold | 0.67 (0.62, 0.72) | Underweight/Normal | 1.00 | Inactive/Insufficient | 1.00 | Never | 1.00 |
| Below poverty threshold | 1.00 | Overweight | 1.09 (1.01, 1.18) | Physically active | 0.93 (0.73, 1.18) | Former | 1.43 (1.30, 1.58) |
|  |  | Obese | 1.39 (1.28, 1.52) |  |  | Current | 1.16 (1.07, 1.24) |
|  |  |  |  |  |  |  |  |
| *Interaction effects*: | F(1, 1118)= 0.19, p= 0.67 | *Interaction effects*: | F(2, 1117)= 0.69, p= 0.50 | *Interaction effects*: | F(1, 1118)= 0.65, p= 0.42 | *Interaction effects*: | F(2, 1117)= 3.61, p= 0.03 |
| Black X At or above poverty threshold | 1.04 (0.87, 1.25) | Black X overweight | 0.92 (0.75, 1.13) | Black X physically active | 1.27 (0.71, 2.26) | Black X former | 0.76 (0.58, 0.99) |
| White X below poverty threshold | 1.00 | Black X obese | 1.03 (0.83, 1.28) | White X inactive/insufficient | 1.00 | Black X current | 0.81 (0.67, 0.96) |
|  |  | White X Underweight/Normal | 1.00 |  |  | White X never | 1.00 |
|  |  |  |  |  |  |  |  |
| Model M: Race X smoking status |  |  |  |  |  |  |  |
| *Main effects*: |  |  |  |  |  |  |  |
| Race |  |  |  |  |  |  |  |
| Black | 0.94 (0.84, 1.04) |  |  |  |  |  |  |
| White | 1.00 |  |  |  |  |  |  |
|  |  |  |  |  |  |  |  |
| Smoking status |  |  |  |  |  |  |  |
| Never | 1.00 |  |  |  |  |  |  |
| Former | 1.35 (1.24, 1.46) |  |  |  |  |  |  |
| Current | 1.82 (1.67, 2.00) |  |  |  |  |  |  |
|  |  |  |  |  |  |  |  |
| *Interaction effects*: | F(2, 1117)= 0.01, p= 0.99 |  |  |  |  |  |  |
| Black X former | 1.00 (0.77, 1.29) |  |  |  |  |  |  |
| Black X current | 1.01 (0.77, 1.33) |  |  |  |  |  |  |
| White X never | 1.00 |  |  |  |  |  |  |
|  |  |  |  |  |  |  |  |

Note: The Kessler Psychological Distress Scale (K6) was used to measure psychological distress, with total self-reported K6 scores of ≥5 out of 24 indicating moderate-severe psychological distress.

**Model A (Interaction Term):** Race x age, adjusting for the rest of the predictors.

**Model B** **(Interaction Term):** Race x sex, adjusting for the rest of the predictors.

**Model C (Interaction Term):** Race x acculturation, adjusting for the rest of the predictors.

**Model D (Interaction Term):** Race x marital status, adjusting for the rest of the predictors.

**Model E (Interaction Term):** Race x region of residence, adjusting for the rest of the predictors.

**Model F (Interaction Term):** Race x employment status, adjusting for the rest of the predictors.

**Model G (Interaction Term):** Race x health insurance status, adjusting for the rest of the predictors.

**Model H (Interaction Term):** Race x education, adjusting for the rest of the predictors.

**Model I (Interaction Term):** Race x poverty status, adjusting for the rest of the predictors.

**Model J (Interaction Term):** Race x BMI, adjusting for the rest of the predictors.

**Model K (Interaction Term):** Race x physical activity, adjusting for the rest of the predictors.

**Model L (Interaction Term):** Race x alcohol drinking status, adjusting for the rest of the predictors.

**Model M (Interaction Term):** Race x smoking status, adjusting for the rest of the predictors.
